# Why are thoracic operations postponed?

**DOI:** 10.1186/1749-8090-7-31

**Published:** 2012-04-11

**Authors:** Georgios I Tagarakis, Christos Voucharas, Vassilios Simopoulos, Dimos Karangelis, Marios E Daskalopoulos, Charalampos Parisis, Apostolos Tsantilas, Ilias Sataitidis, Stefania Lampoura, Georgios Vretzakis, Nikolaos B Tsilimingas

**Affiliations:** 1 Department of Cardiovascular and Thoracic Surgery,, University of Thessaly, Larissa, Greece; 2 Anaximandrou 24, 54250 Thessaloniki, Greece

## Abstract

**Aim:**

To investigate and present the reasons that cause the postponement of thoracic surgical operations.

**Methods:**

We retrospectively included in the study all patients submitted to elective thoracic surgery in our department during the 4-year period 2007-2010 and noted all cases of postponement after official inclusion in the operating schedule.

**Results:**

81 out of a total of 542 patients (14.9%) scheduled for elective thoracic operation had their procedure postponed. The reasons were mainly organisatory (in 42 cases, 51.85%), which in order of significance were: shortage in matching erythrocyte units, shortage in anaesthetic/nursing staff and unavailability in operating rooms. The rest of the cases (39, 48.1%) were postponed due to medical reasons, which in descending order of significance were: respiratory infections and exacerbations of COPD, cardiological problems, misregulation of antiplatelet/antithrombotic drugs and infections from other systems (gastrointestinal, urinary, etc.). Elderly male patients planned for major/oncologic surgery were most possible to have their operation postponed for medical reasons.

**Discussion-Conclusions:**

Thoracic operations are postponed owed to organisatory as well as medical reasons, the latter mainly affecting elderly, morbid patients awaiting for major/oncologic surgery.

## Introduction

With minor exceptions, operations belonging to the field of thoracic surgery constitute demanding procedures, sometimes stressful even for experienced surgeons. Most importantly, a procedure performed in this area of vital organs provokes a sense of anxiety and insecurity for the patients themselves, who are also often psychologically burdened by serious comorbidities, as well as by a whole series of preoperative diagnostic tests and procedures. In this setting, the postponement of the operation for any reason, organisatory or medical, causes a great amount of extra stress, creating a psychological condition which sets obstacles to the flawless perioperative course of the patient [1,2]. In addition, and in some rare cases of malignant disease, the delay in performing the operation can have devastating results through alterations of the staging and prognostic parameters of the disease.

Herein, we are presenting the 4-year experience of our department on the subject, in an attempt to elucidate the reasons for the postponement of thoracic procedures, so as to avoid to the most possible extent similar mishappenings in the future.

## Methods

We retrospectively included in this epidemiologic analysis all patients prepared and scheduled for elective thoracic surgery in our department in the 4-year period 2007-2010, whose operation was postponed for any reason. We excluded from the study all patients submitted to urgent surgery, such as surgery for thoracic trauma or superior vena cava syndrome. The aforementioned criteria led to the inclusion in the study of 542 out of a total of 613 thoracic surgical patients (88.41%) operated in our department in the aforementioned period.

In regard to the characteristics of our department: it is a cardiovascular and thoracic surgery department of a university tertiary care hospital, operating with European Union standards, covering with every-day 24-hour duty an area of responsibility of 1.5 million inhabitants in Central Greece.

### Statistical analysis

For the statistical analysis the *χ*2 criterion was applied for categorical parameters, the t-student (unpaired) for continuous ones. The Kolmogorov-Smirnoff test was used for the evaluation of normal distribution of samples. The level of statistical significance was set at a level for *p *< 0.05.

## Results

In the examined period 81 patients (81/542 = 14.9%) had their elective thoracic operation postponed. In 42 of the cases (51.8%) the main reason was organisatory, more specifically: i) in 20 cases (47.6%) there was shortage in matching erythrocyte units, in 18 cases (42.8%), there was lack in anaesthetic and nursing staff and in 4 cases (9.5%) there was unavailability in operating rooms (Table 1).

**Table 1:** Analysis of organisatory problems and medical conditions responsible for postponement of elective thoracic surgical operations

Organisatory, n = 42 (51.8%)	Medical n = 39 (48.1%)
	
	15 patients (38.4%) respiratory infections/exacerbations of CABG
20 patients (47.6%) shortage in matching erythrocyte units	9 patients (23%) cardiological problems (23%)

18 patients (42.8%) shortage in anaesthetic/nursing staff	8 patients (20.5%), misregulation of antiplatelet/antithrombotic drugs

4 patients (9.5%) unavailability in operating rooms	7 patients (17.9%) febrile conditions/infections of gastrointestinal and urinary tract

The table shows all organisatory and medical reasons that lead to the postponement of thoracic surgical operations

The rest 39 patients (48.1%) had their procedure postponed for medical reasons (Table 1). These were noted in descending order of frequency as follows: respiratory infections and COPD exacerbations 15 (38.4%), cardiological problems 9 (23%), misregulation of antiplatelet/antithrombotic drugs 8 (20.5%), other infections/febrile conditions (from gastrointestinal and urinary tract) 7 patients (17.9%)-(Table 1).

The most prominent features of patients whose procedure was postponed for medical reasons were: advanced age (mean age 69.4 vs 59.8, *p *< 0.01, male gender 82% vs 69.9%, *p *< 0.01 and programming for major/oncolocgic surgery 74.3% vs 45.5%, *p *< 0.01). These data are depicted in detail in Table 2.

**Table 2:** Comparative analysis of demographic and procedure-related parameters between patients with operations postponed due to medical reasons and the rest of the patients

	Patients postponed for medical reasonsn = 39	Rest of the patientsn = 503	Statistical Significance
Age	69.4	59.8	p < 0.01

Gender (male)	32 (82%)	352 (69.9%)	p < 0.01

Years of education	7.6	7.5	non significant

Scheduled for major/oncologic surgery (lobectomy, bilobectomy, pulmonectomy)	29 (74.3%)	229 (45.5)	p < 0.001

Scheduled for minor/surgery for benign disease (biopsies, bullectomy/pleurodesis)	10 (25.64%)	271 (53.87%)	p < 0.01

The table shows comparative analysis of certain parameters between the group of patients whose operation was postponed for medical reasons and the rest of the patients. The *x*2 criterion was applied for categorical parameters, the t-student (unpaired) for continuous ones. The Kolmogorov-Smirnoff test was used for the evaluation of normal distribution of samples. The level of statistical significance was set at a level for *p *< 0.05

## Discussion

This study deals with the important issue of postponement of thoracic operations, a mishappening that causes both psychological stress to the patients as well as enhanced hospitalization costs for the related health providing organization [1,2]. To the best of our knowledge this is the first study of the kind in medical literature regarding thoracic patients and one of a few dealing with the subject of postponement of surgical procedures in general [3-6]. An interesting study by Lowthian et al. [7], which retains its value regardless of the fact that it was not confined to thoracic surgical patients, has shown that rationalized planning and thorough preoperative preparation may bring the percentage of postponement for elective surgical procedures as low as 1%. Our study is also targeting to this direction; its main points of interest are analysed right away.

The main reasons of postponement were organisatory; these included unavailability in matching erythrocyte units, shortage in anaesthetic/nursing staff and unavailability in operating rooms.

Unavailability in matching erythrocyte units can be explained by the fact that our hospital also handles urgent cases and sometimes the units secured for the thoracic patients must be given to patients submitted to surgery for acute-life-threatening conditions.

Unavailability in anaesthetic/nursing staff and operating rooms can be justified by the responsibility of the hospital and our department for a greater geographical area and the burden caused by acute and urgent cases. However, the basic background for this issue lies in the economic recession noted in Greece, as well as in other countries of the European Union, which has as a result major financial cuts which affect overtime payments and hiring of required personell.

Morbid patients with history of COPD and cardiological problems, mostly with advanced age and belonging to the male gender, are the ones most frequently having their thoracic procedure postponed. Another interesting finding is that among cases of postponement for medical reasons, patients planned for major/oncologic surgery (lobectomy, bilobectomy, pulmonectomy) have their operation more usually postponed than patients planned for minor or surgery for benign disease, such as biopsies, or bullectomy and pleurodesis. This can probably be explained both by the fact that patients with sever oncological problems often also face other comorbidities, as well as by the fact that patients planned for major surgery generally have the need for more thorough and meticulous preoperative preparation.

We hope that the aforementioned conclusions will assist thoracic surgeons, anaesthesiologists and nursing staff dealing with this important category of patients in avoiding such cases of postponement in the future.

## Conflicts of interests

The authors declare that they have no competing interests.

## Authors' contributions

GT was the main author. CVperformed data research, literature research and checked the paper. VS has performed literature research and checked the paper. DK has performed literature research and checked the paper. MD performed literature research and checked the paper. CP has examined most of the patients as an attending cardiologist and checked the paper. AT assisted in the statistical analysis. IS was a member of the team and checked the paper. SL checked the paper. GV was a member of the anaestaetic team and checked the paper. NT was the chief surgeon, head of the department and had the overall supervision for the study. All authors have read and approved the final manuscript.
